# Synthesis of Human Milk Oligosaccharides: Protein Engineering Strategies for Improved Enzymatic Transglycosylation

**DOI:** 10.3390/molecules24112033

**Published:** 2019-05-28

**Authors:** Birgitte Zeuner, David Teze, Jan Muschiol, Anne S. Meyer

**Affiliations:** Protein Chemistry and Enzyme Technology, Department of Biotechnology and Biomedicine, Technical University of Denmark, 2800 Kgs Lyngby, Denmark; bzeu@dtu.dk (B.Z.); davtez@dtu.dk (D.T.); jmus@dtu.dk (J.M.)

**Keywords:** human milk oligosaccharides, transglycosylation, protein engineering, fucosidase, sialidase, β-*N*-acetylhexosaminidase, transfucosylation, transsialylation, casein glycomacropeptide

## Abstract

Human milk oligosaccharides (HMOs) signify a unique group of oligosaccharides in breast milk, which is of major importance for infant health and development. The functional benefits of HMOs create an enormous impetus for biosynthetic production of HMOs for use as additives in infant formula and other products. HMO molecules can be synthesized chemically, via fermentation, and by enzymatic synthesis. This treatise discusses these different techniques, with particular focus on harnessing enzymes for controlled enzymatic synthesis of HMO molecules. In order to foster precise and high-yield enzymatic synthesis, several novel protein engineering approaches have been reported, mainly concerning changing glycoside hydrolases to catalyze relevant transglycosylations. The protein engineering strategies for these enzymes range from rationally modifying specific catalytic residues, over targeted subsite −1 mutations, to unique and novel transplantations of designed peptide sequences near the active site, so-called loop engineering. These strategies have proven useful to foster enhanced transglycosylation to promote different types of HMO synthesis reactions. The rationale of subsite −1 modification, acceptor binding site matching, and loop engineering, including changes that may alter the spatial arrangement of water in the enzyme active site region, may prove useful for novel enzyme-catalyzed carbohydrate design in general.

## 1. Introduction

**Figure 1:**
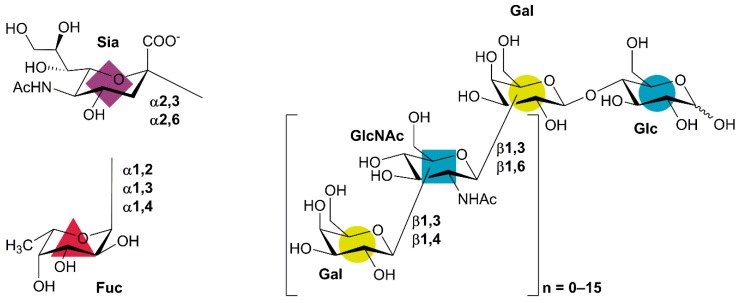
Human milk oligosaccharide (HMO) blueprint structure [1]. Gal: galactose, Glc: glucose, GlcNAc: *N*-acetylglucosamine, Fuc: fucose, Sia: sialic acid (*N*-acetylneuraminic acid). Lactose is at the reducing end of all HMO structures, which may be elongated with β-*N*-acetyllactosamine (LacNAc) or lacto-*N*-biose units. Both lactose and elongated structures may be decorated with Fuc and/or Sia. The colored shapes indicate the Symbol Nomenclature for Glycans (SNFG [8], https://www.ncbi.nlm.nih.gov/glycans/snfg.html), which is commonly used for presenting the numerous HMO structures.

Human milk oligosaccharides (HMOs) denote a group of lactose-based carbohydrate structures in human breast milk, which are considered to exert health benefits on the breast-fed baby via various mechanisms. HMOs are present in human milk at concentrations of 5−15 g/L, which makes HMOs an abundant component of human milk [1]. In contrast, the concentration and the variety of HMO-identical structures are much lower in bovine milk, which is the basis of infant formula [2,3]. HMOs are critically important for early development and infant health since they function as prebiotics and antimicrobial agents in the gut of breastfed infants. They further protect the infant against pathogens by functioning as soluble decoy receptors for pathogen adhesion as well as through a number of immunomodulating effects [1,4]. In a recent study, purified HMOs have also been shown to exert beneficial effects in adults [5], thus widening the potential applications and business opportunities of industrially produced HMOs. No single HMO has all these effects alone, suggesting different roles for the over 150 different HMO structures that have been identified in human milk [6,7]. All HMO structures are variations of the specific HMO blueprint pattern composed of (up to) five different monosaccharides, always in their pyranose form and in the same anomeric configuration: β-d-galactose (Gal), β-d-glucose (Glc), β-d-*N*-acetyglucosamine (GlcNAc), α-l-fucose (Fuc), and the sialic acid α-d-*N*-acetylneuraminic acid (Sia) (Figure 1). The reducing end of HMOs is lactose (Lac; Gal-β1,4-Glc). Lac can be elongated at the Gal *O*-3 with lacto-*N*-biose (Gal-β1,3-GlcNAc), which prevents further elongation. The Gal moiety of Lac can also be elongated by β-*N*-acetyllactosamine (LacNAc; Gal-β1,4-GlcNAc) units either at *O*-3 or *O*-6, opening it up for further extensions. Finally, Gal, Glc, and GlcNAc residues can be fucosylated or sialylated: Gal with Fuc at *O*-2 or *O*-3 as well as with Sia at *O*-3 and *O*-6, GlcNAc with Fuc at either *O*-3 or *O*-4 and with Sia at *O*-6, and Glc only by Fuc at *O*-3 (Figure 1) [1]. In summary, five different monosaccharides and 10 different types of linkages make up the entire plethora of HMO structures. The specific blueprint patterns make it possible to achieve synthesis of a range of HMO structures through targeting just a few different enzymatic activities either in vivo or in vitro.

While over 150 HMO structures exist, only 2′-fucosyllactose (2′-FL) and lacto-*N*-neotetraose (LNnT) are currently commercially available for addition to infant formula [9]. Microbial engineering work has recently made it possible to produce these two compounds in industrial scale by fermentation of genetically modified *Escherichia coli* [9,10]. Several years of work on commercialization and regulatory approval of synthesized HMOs has now paved the way for expanding the HMO portfolio for future innovative food products beyond infant formula [9,10]. 2′-FL was an obvious starting point for HMO production as it is the most abundant HMO [11,12] and has a simple structure. In contrast, LNnT is less abundant both on its own and as an HMO core, and is also present in human milk in lower levels than, e.g., lacto-*N*-tetraose (LNT) [11,12], but it appears that LNnT is easier to synthesize in large scale and was therefore marketed first [9,13,14]. Indeed, for more complex and larger structures, fermentation yields are often low [13,15].

Based on currently available in vitro and in vivo studies, it is likely that the putative synergistic effect of numerous different HMOs can provide additional benefits in terms of health maintenance and microbiota composition of infants and adults [4,5,16,17]. Thus, it is crucial to include as many different HMO structures as possible in research of their bioactivity and health effects as well as in food supplementation. Two recent studies have indicated that the more complex fucosylated and sialylated HMOs had a larger antimicrobial effect on certain group B *Streptococcus* strains than fucosylated or sialylated lactose, and that the location and degree of fucosylation and sialylation play a key role in the antimicrobial activity of HMOs [16,17].

Currently, many HMOs are not available in sufficient quantities and there is no efficient route to their production. The status of enzymatic HMO production—both in vitro and in cell factories—was recently thoroughly reviewed [15], and while great progress has been made over the past two decades, the discrepancy between human milk composition and currently obtainable HMOs is evident, providing a continued impetus to produce a wider span of true HMO structures by controlled enzymatic synthesis. One way to accomplish production of larger and more complex HMOs is to employ transglycosylation catalyzed by glycoside hydrolases (or glycosidases, GHs), possibly in combination with use of fermentation-derived backbone structures such as LNnT or LNT as acceptor substrates for the enzymatic glycosylation. This review summarizes the various HMO production methods and focuses particularly on glycosidase-catalyzed transglycosylation, since this technology appears to be the most promising to complement microbial cell factories (the current industrial technology for production of a few HMO structures) in the quest to expand the industrial production to cover the majority of the naturally occurring HMO portfolio. Glycosidases are important enzymes for industrial-scale glycan synthesis due to their ability to use naturally occurring glycans, e.g., from major agro-industrial side streams, as glycosyl donor substrates. An example of current industrial use of glycosidases for transglycosylation is the production of prebiotic fructo-oligosaccharides (FOS; the basic structure is a terminal Glc unit α1,2-linked to a linear chain of two or more β2,1-linked fructose moieties) and galacto-oligosaccharides (GOS; a family of structures comprising two or more Gal units linked by β-glycosidic bonds, often with a terminal Glc moiety) [18,19,20]. Today, both FOS and GOS are added to infant formula, although they are not true HMOs and have been classified as unnecessary in infant formula [10]. This review focuses on reports of enzymatic HMO synthesis from abundant natural substrates. A major focal point of this review is different strategies to improve enzymatic transglycosylation through rational protein engineering, even if the synthesized products are non-HMO oligosaccharides. Protein engineering to attain enhanced transglycosylation is currently manifesting itself as a valuable tool for improving enzyme performance in transglycosylation reactions and a crucial means of obtaining feasible processes.

## 2. Routes to HMO Production Outside the Mammary Gland

While breast milk donation programs exist as part of health care services, especially for premature infants, this natural source can by no means cover the demands of the industry seeking to add HMOs to infant formula in order to supplement formula-fed infants with these beneficial components. Instead, various technologies are in play in pursuit of a viable industrial process for HMO production: fermentation of microbial cell factories, chemical synthesis, and a number of different enzymatic in vitro reactions.

### 2.1. Microbial Cell Factories

In 2015 and 2016, the first two HMOs were marketed in the US and Europe in an intense competition between American Abbott Laboratories, German Jennewein Biotechnologie GmbH, and Danish Glycom A/S [9,10]. Following decades of research into metabolic engineering, 2′-FL and LNnT were the first HMOs to become available in industrial scale from fermentation of engineered *E. coli*. Both compounds have been registered as safe with the U.S. Food and Drug Administration (FDA) and the European Food Safety Authority (EFSA) [9,13] and are now available in infant formula in more than 30 countries. The research developments leading to this breakthrough as well as the challenges faced were recently reviewed by authors from HMO-producing companies [9,10]. Fermentation titers up to 180 g/L have been reported for 2′-FL [9]. Infant formula containing 2′-FL and LNnT was safe and well-tolerated in a clinical trial with infants of up to 6 months; parents reported less morbidity upon ingestion of 2′-FL and LNnT [21]. Beneficial effects on adult microbiota have also been reported [5]. Other HMO structures which can be produced in industrial scale by fermentation, but are not yet marketed in infant formula, are LNT and difucosyllactose (DFL) [9]. Additionally, efforts have been made to engineer *Saccharomyces cerevisiae* [22] or *Lactococcus lactis* [23]—organisms having the GRAS (generally regarded as safe) label—to produce HMOs.

However, while metabolic engineering and fermentation technology has decisively improved HMO production within the past two decades, the technology also faces limitations. For more complex HMOs, fermentation titers are often low and/or the products remain intracellular [13,15]. For industrial production, extracellular products significantly ease purification [9]. In addition, a suitable β1,6-*N*-acetylglucosaminyltransferase to produce branched HMOs is still missing [13]. Branched HMO structures comprise a large part of the natural HMO pool [11], but in fermentation processes, they have only been obtained in low titers and mainly inside the cell as a side product where the *Neisseria meningitides* β1,3-*N*-acetylglucosaminyltransferase catalyzed formation of the β1,6-linkage [15,24]. A human β1,6-*N*-acetylglucosaminyltransferase expressed in human embryonic kidney cells (HEK293) has been employed for in vitro chemoenzymatic synthesis of branched HMOs [25] (see Section 2.3), but its use for production by fermentation has yet to be reported.

### 2.2. Chemical Synthesis

More than 15 different HMO structures have been prepared by chemical synthesis [13,26]. Examples include lab-scale synthesis of lacto-*N*-fucopentaose (LNFP I) [26], gram-scale synthesis of LNT [27], and a recent report of kilogram-scale synthesis of 2′-FL [28]. The main challenge in chemical synthesis of HMOs is the high number of protecting group manipulations, which further increases with chain length and branching. As a result, chemical synthesis is often time-consuming and gives low product yields. Furthermore, chemical synthesis involves toxic reagents and usually transition metal catalysts [29]. Consequently, chemical synthesis of complex carbohydrates is often not a cost-efficient production method for large-scale synthesis of HMOs. Lowering the number of required intermediates and product purification steps is a key to obtaining a feasible, scalable process [28]. Although chemically synthesized 2′-FL and LNnT were originally registered for use as novel ingredients for infant formula [10], the use of microbial cell factories is currently the only method applied for industrial production of these HMOs, being a more economically feasible method [9]. Nevertheless, chemical synthesis remains an important tool for analytical purposes as well as for generation of building blocks for chemoenzymatic synthesis methods. Indeed, to fully harvest the potential of chemical synthesis for production of HMOs, it is now frequently combined with enzymatic synthesis, particularly using regio- and stereospecific glycosyltransferases (see Section 2.3) [25,30,31,32]. Recently, a chemoenzymatic route using a glycosidase for synthesis of the HMO precursor structure lacto-*N*-triose II (LNT2; GlcNAc-β1,3-Lac) was also reported [33], and the same approach was used for synthesis of LNT [34]. Such chemoenzymatic approaches are particularly useful for generating large libraries of HMO structures, which can be used for bioactivity studies [25,30,35].

### 2.3. Enzymatic Synthesis in Vitro

In humans, HMOs are synthesized by Leloir glycosyl transferases (GTs), i.e., GTs active on sugar–nucleotide donor substrates. GTs are usually highly regio- and stereospecific, thus enabling efficient and precise glycoside synthesis in a single reaction. As outlined in Section 2.1, these enzymes are successfully utilized for HMO synthesis using microbial cell factories. However, the use of GTs in vitro is considered more difficult. Indeed, their requirement of sugar–nucleotide substrates requires the setup of multienzyme cascade systems for nucleotide recycling to increase the process cost-efficiency. Furthermore, GTs can be hard to express with adequate yields. These challenges and the current opportunities for development of GTs into efficient biocatalysts were recently reviewed elsewhere [36]. For HMO synthesis, several examples of (one-pot) multienzyme cascade systems including GTs exist. Sialyltransferases (SiaTs) of varying specificity have been employed for synthesis of sialylated HMOs such as 3′-sialyllactose (3′-SL), 6′-sialyllactose (6′-SL), and disialyllacto-*N*-tetraose (DSLNT) [37,38,39]. The Korean company GeneChem uses SiaTs for synthesis of 3′-SL and 6′-SL in large scale and had their 3′-SL GRAS-approved in 2018 [9,37,40,41]. Certain sialyltransferases are dual-activity enzymes, which also exhibit transsialidase activity and thus accept non-nucleotide donors [42,43,44,45]. This feature has, however, not been described for any other HMO-relevant GTs. Recently, a comprehensive library of 60 different HMO structures was synthesized by a series of human GTs expressed in a mammalian cell line [25]. Combined with chemical synthesis to equip the core lactose with a multifunctional anomeric linker, a microarray was created and utilized for assessing protein binding [25]. Similarly, a library of defined linear HMO structures was synthesized recently using a set of microbial and human GTs [46]. These are excellent examples of how GTs are particularly strong for synthesis of clearly defined HMO structures of varying length with different branching, fucosylation, and sialylation patterns for analytical purposes and especially for bioactivity studies. However, such reactions are currently not scalable to industrial production levels [10].

Glycoside hydrolases (GHs) present an alternative to the sugar–nucleotide dependent GTs for catalysis of HMO synthesis. In contrast to GTs, GHs accept cheap and abundant substrates and the enzymes are often robust and easy to express. The main challenge of using GHs for transglycosylation is their inherent hydrolysis activity, usually of both substrates and products. While several examples of naturally occurring transglycosidases exist [47], i.e., GHs which essentially do not catalyze hydrolysis, the only HMO-relevant example is the transsialidase from *Trypanosoma cruzi*, TcTS [48]. Consequently, our focus is turned to the GHs, which catalyze transglycosylation in competition with hydrolysis. Reaction conditions can be optimized to favor transglycosylation, e.g., through increasing substrate concentration or by adjusting pH [49,50]. However, the strongest tool to efficiently improve transglycosylation activity and/or diminish hydrolytic activity in GHs is protein engineering. Enzymatic transglycosylation has shown great potential in HMO synthesis, especially for sialylation or fucosylation of lactose or LNT [42,48,51,52,53,54,55,56,57], but also for synthesis of HMO core structures [58,59,60,61]. This technology holds great potential to expand the current limited industrial-scale HMO portfolio, but much of this potential relies on either the discovery of novel transglycosidases or protein engineering of the enzymes for improved transglycosylation efficiency.

## 3. Glycosidase-Catalyzed Transglycosylation

**Figure 2:**
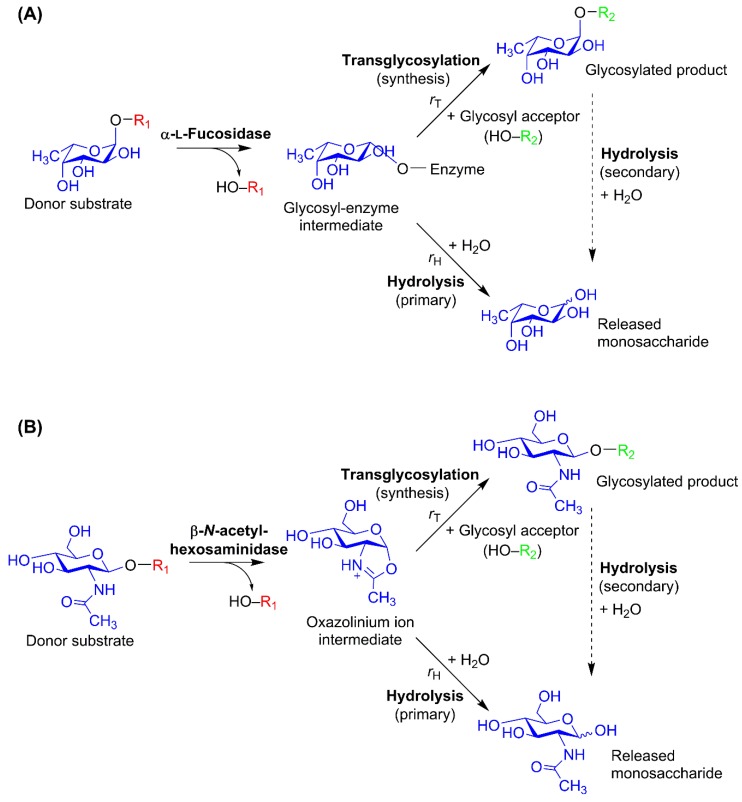
Reaction scheme sketches for glycosidase-catalyzed transglycosylation, which takes place in competition with substrate hydrolysis [49,68]. (**A**) Classical Koshland double-displacement mechanism exemplified by the α-l-fucosidase reaction: The intermediate, which is in the opposite anomeric configuration compared to the substrate and product as per the double displacement mechanism of retaining glycoside hydrolases (GHs), is attacked by a nucleophile. If this nucleophile is water, (primary) hydrolysis occurs. If a glycosyl acceptor performs the nucleophilic attack, transglycosylation occurs. (**B**) Substrate-assisted reaction mechanism of the GH20 β-*N*-acetylhexosaminidases [65]. For both reaction mechanisms, the resulting glycosylated product may also be subject to (secondary) hydrolysis catalyzed by the same glycosidase. The balance between the transglycosylation rate (*r*_T_) and the hydrolytic rate (*r*_H_) is governed by the reaction conditions as well as by enzyme properties, which can be altered through protein engineering. Regioselectivity in the product formation may vary. In HMO synthesis, R_1_ and R_2_ are glycosides, but for transglycosylation, in general, they can be other compounds, e.g., primary alcohols.

Enzymatic transglycosylation is catalyzed by retaining glycoside hydrolases (GHs), i.e., glycosidases, which retain the configuration of the anomeric center of their products. The active site of a GH is described by a subsite nomenclature [62]: subsites are labelled from −*n* to +*n*, where *n* is an integer. While −*n* represents the nonreducing end of the glycoside recognized by the GH, +*n* represents the reducing end. Catalytic cleavage takes place between the −1 and +1 subsites. For HMO synthesis, exo-acting GHs with a single negative subsite (−1) are most common, but disaccharide-transferring GHs such as lacto-*N*-biosidases [61] are also relevant, and in general, transglycosylation is not limited to exo-acting enzymes [47]. To produce oligosaccharides through kinetically controlled transglycosylation, the glycosyl moiety to be transferred to an acceptor substrate must be linked with a glycosidic bond in the donor substrate (Figure 2) [63]. This donor glycosyl moiety is bound in the negative subsite(s) and usually defines the names of the GHs which recognize it. With the exception of most GlcNAc/GalNAc-processing enzymes, retaining GHs operate with the classical Koshland double-displacement mechanism, which is a two-step reaction with at least two transition states [47,64]. In the first step—the glycosylation step—a covalent glycosyl–enzyme intermediate is formed upon binding of the donor glycosyl in the active and release of a leaving group; this takes place via an oxocarbenium-like transition state. This intermediate has the opposite anomeric configuration to that of substrate and product, and in the second step—the deglycosylation step—it undergoes nucleophilic attack from either water or a glycosyl acceptor. Through the second transition state, this nucleophilic attack results in either hydrolysis or transglycosylation, depending on the nature of the nucleophile (Figure 2A). The retaining GH mechanism, its rate constants, and its importance in GH engineering has been thoroughly described elsewhere [47]. The GH20 β-*N*-acetylhexosaminidases employ a substrate-assisted reaction mechanism, where the 2-acetamido group of the substrate acts as an intramolecular nucleophile and the GlcNAc forms an oxazolinium ion intermediate rather than a glycosyl–enzyme intermediate (Figure 2B) [65].

It is evident that transglycosylation takes place in competition with hydrolysis (Figure 2). The balance between the transglycosylation rate (*r*_T_) and the hydrolysis rate (*r*_H_) is largely governed by enzyme properties, which can by modified by protein engineering. However, transglycosylation can also be favored through reaction conditions such as water activity, pH, temperature, and substrate concentrations [49,50]. For HMO synthesis, the most common trick is the use of high acceptor substrate concentration. A high acceptor-to-donor ratio (A:D) can make many retaining GHs catalyze transglycosylation with moderate yields [53,66], but to work well at equimolar ratios, protein engineering is often preferable to reach appreciable product yields [55,67]. Not only the donor substrate, but also the transglycosylation product may be subject to hydrolysis catalyzed by the same GH that catalyzed its formation (secondary hydrolysis; Figure 2). This leads to a transient product maximum, and in a case where product hydrolysis is pronounced, tight reaction time control is essential. Thus, successful protein engineering is tightly linked to reduction of hydrolytic activity.

While GHs are stereospecific, as they produce products with defined anomeric configuration, regioselectivity varies between enzymes and may depend on acceptor structure. Low regioselectivity can be observed both as several different hydroxyl groups on the same monosaccharide moiety of the acceptor and/or as hydroxyl groups from different monosaccharide moieties acting as nucleophiles [53,59,66,69]. Enzyme regioselectivity must be harnessed when employing glycosidases for HMO synthesis, either by choosing highly regioselective enzymes [55], through process design to remove regioisomers either by specific enzymatic degradation [61,70] or by purification, or through protein engineering for improved regioselectivity [70,71,72,73], which is beyond the scope of this review.

## 4. Improved Transglycosylation through Protein Engineering

Over the last decade, protein engineering has been established as a strong tool for improving the transglycosylation efficiency of HMO-relevant glycosyl hydrolases [54,55,59,67,73,74,75,76,77]. We also review a few approaches to improve transglycosylation efficiency that have not yet been reported for HMO synthesis but could be relevant for the future of the field.

### 4.1. Glycosynthases

The most generic strategy to turn GHs into synthetic tools has so far been a mechanism-based approach resulting in so-called glycosynthases; this approach was recently reviewed elsewhere [78]. It consists of mutating the catalytic nucleophile (or one of the catalytic residues, in the case of substrate-assisted mechanisms) [79] and providing an activated donor of the same anomeric configuration as the reaction intermediate, i.e., the opposite configuration of the desired product [80]. The requirement for activated donors (typically fluoride, sometimes azido or oxazoline saccharide derivatives) limits their use in industrial scale. Regarding the glycosidic bonds to be formed in HMOs, successful reports of galactosynthases [81] and fucosynthases [82,83,84,85] exist. For fucosynthases, an issue is the fact that β-fluoride donors are much less stable than α-fluoride donors [78]. Instead, sufficient substrate stability was achieved with a β-fucosyl azide donor substrate [82]. For glycosynthases which accept GlcNAc donors, lessons learnt with chitinases [86,87] and endo-β-*N*-acetylglucosaminidases [88] may be transferable to HMO structures. However, a recent study on a GH20 β-*N*-acetylhexosaminidase showed that the general strategy of glycosynthase engineering was not applicable [89]. Glycosynthase mutants of a GH20 lacto-*N*-biosidase did not outperform the wild-type enzyme in terms of yield or ratio between transglycosylation and hydrolysis. The only advantage of this glycosynthase was the significantly lowered product hydrolysis rate [34]. No sialyl-transferring glycosynthase has been reported. Notably, glycosynthase-catalyzed reactions lead to anionic byproducts such as azide and fluoride that prevent their use in food production. Despite their high transglycosylation product yields, glycosynthases may struggle with the same regioselectivity issues as encountered by their parent glycosidases [82,90]. In some cases, equally good or better transglycosylation yields were obtained with engineered glycosidases compared to the corresponding glycosynthases [67,75,91].

### 4.2. Rational Design

Unlike the glycosynthase strategy, alternative approaches for improved transglycosylation efficiency maintain the GH activity on natural substrates. Three common approaches can be outlined: (1) to modify the −1 subsite in order to reduce the transition states stabilization of the catalyzed reactions (particularly hydrolysis); (2) to increase the affinity in the acceptor binding site(s), commonly subsites +1 and +2; or (3) to disrupt binding of catalytic water [50]. While these are the goals, translating them into identification of specific amino acid mutation targets is not necessarily straightforward. In the following, available strategies are introduced.

The CAZy database divides GHs into families based on sequence similarity (www.cazy.org) [92]. Thus, for GHs within the same family, it may be possible to extrapolate successful mutations, although this is not a given [55,75,93,94]. Indeed, mutations in the negative subsites are more likely to be transferable than mutations in positive subsites, as the positive subsites may differ substantially within each family. However, examples of transferability for positive subsite mutations do exist [93]. If working with a well-studied family of enzymes where prior successful engineering studies and structural data are available, rational design is possible. Certain GH families contain both hydrolases and natural transglycosidases, which can be used as templates for rational engineering of the hydrolases [47,74]. Moreover, hydrophobic platforms are obvious targets in all subsites, as several studies have indicated the importance of aromatic residues for increasing the ratio of transglycosylation over hydrolysis (T/H) [66,67,93,95,96,97,98,99]. However, when aiming to conquer new territory, such templates or previous successes are often not available. In addition, the changes that lead to increased transglycosylation efficiency are often minute and hard to pinpoint from a structural point of view [67,98], or rationalization of the previously obtained results may be unsuccessful [67,95]. In many cases, molecular modeling, molecular dynamics, and quantum mechanics/molecular mechanics (QM/MM) simulations are tentatively used to rationalize the obtained results for future predictions [50,100,101,102,103,104]. However, the understanding of the structure–function relationships governing the transglycosylation/hydrolysis balance in an enzyme is still so far from complete that generic *in silico* methods for predicting successful mutations belong to the future. The currently available (semi)generic (semi)rational approaches to engineer glycosidases for improved transglycosylation are outlined below.

#### 4.2.1. Targeting Conserved Residues in Negative Subsite(s)

A stark difference in rates commonly discriminates hydrolases and natural transglycosidases, the former being much faster [99,105,106]. Hence, a logical step to increase transglycosylation yields by protein engineering could be to seek enzymes with decreased hydrolysis. Without any knowledge of sequence or structure, one can use directed evolution where diversity is generated by error-prone PCR or gene shuffling and screening for reduced hydrolysis. The screening task is large in directed evolution, but it can be accomplished with direct colorimetric screening on petri dishes using the classical method with X-Gal (5-bromo-4-chloro-3-indolyl β-d-galactopyranoside) or its analogues [75,90,107]. Taking advantage of the fact that good transglycosidases have higher activity in the presence of an acceptor substrate, a colorimetric screen measuring apparent rates of both hydrolysis and transglycosylation directly on colonies have been developed [108]. Direct detection of transglycosylation products in large libraries could also be done using biosensor strains expressing green fluorescent protein (GFP) upon presence of specific HMO molecules [109], but this strategy has not yet been applied for enzyme engineering purposes.

Outcomes of directed evolution have repeatedly singled out the −1 subsite as a common location for mutations that drastically increased transglycosylation yields. The mutated residues also appeared to be often conserved through evolution at the sequence level [75,108,110]. Consequently, targeting conserved residues in subsite −1 was proposed as a semirational approach for increasing T/H. It was hypothesized that such mutations decrease transition states (TS^‡^) stabilization, and that this will usually affects the hydrolysis TS^‡^ more than the transglycosylation one. A modification of TS^‡^ stabilization of a given reaction directly translates into a rate change of said reaction. The resulting mutant enzymes thus have an increased T/H ratio, although often at the expense of catalytic efficiency [67]. The systematic mutagenesis of conserved residues in the −1 subsite was applied to a GH1 β-glycosidase, where the mutation of any of seven first-shell residues led to improved transglycosidases (65–82% yields of disaccharide synthesis versus 36% for the native enzyme) [67]. This approach was then refined on a GH36 α-galactosidase, where it was shown that second-shell conserved residues were important targets, and that “conservative” mutations (e.g., Tyr into Phe) preserved higher overall activity than Ala mutants and preserved or increased synthetic yields [95]. Another implementation, beyond HMO synthesis, allowed high transglycosylation yields (80%) on a GH51 α-arabinofuranosidase for a combination of conserved residues from the −1 subsite and *in silico* screening for binding affinity in the acceptor subsites [111]. Although not all mutations result in mutants with improved transglycosylation capacity, this strategy dramatically lowers the screening effort compared to directed evolution or site-saturation mutagenesis of active site residues. Besides reducing the sequence space to pinpoint good candidates, an advantageous feature of conserved residues modification lies in its transferability: once a particular mutation has been identified as beneficial for TG yields, it can be transposed on related enzymes, i.e., enzymes within the same GH family or clan. This “mutation grafting” has been successful in a number of cases [112,113], from which we would highlight the transfer of mutations identified in the *Thermotoga maritima* GH29A α-fucosidase to a GH29B α-1,3/4-fucosidase from *Bifidobacterium longum subsp. infantis* to synthesize fucosylated HMOs [55].

#### 4.2.2. Loop Engineering

Another recently emerged strategy targets loops close to the active site, which are either inserted or exchanged in order to shield the active site from water or to alter the water network inside it. The strategy relies on structural data for loop identification. At best, crystal structures are available for the targeted enzyme or at least within the same GH family in order to allow homology modeling. Alternatively, secondary structure prediction tools could be used in combination with multiple sequence alignment (MSA). Three different examples of loop engineering of HMO-relevant enzymes exist; together, they outline two different strategies to identify relevant loops.

The human pathogen *Trypanosoma cruzi* expresses a native GH33 transsialidase, TcTS, which efficiently catalyzes formation of α2,3-sialosides. Nonpathogenic *Trypanosoma rangeli* expresses a GH33 sialidase, TrSA, which has 70% sequence identity to TcTS, but does not catalyze transsialylation. Consequently, TcTS has been used several times as a template for engineering TrSA into an efficient transsialidase [52,74,99]. One of these engineering studies used a rational strategy where only amino acids up to 14 Å from the active were considered, particularly targeting amino acids in the sequence alignment with large chemical differences, proposing that they relate to a low probability of random evolutionary substitution [52]. With this approach, a seven-amino-acids-long loop almost 14 Å from the active site was identified: exchanging these seven amino acids with the corresponding sequence from TcTS introduced a net charge of +3. Since the loops aligned perfectly in the crystal structures of TrSA and TcTS irrespective of ligand binding, it was hypothesized that the positive effect of this loop exchange on the hydrolytic activity (4-fold decrease) was due to a reversal of the water network in the active site. It was hypothesized that the low hydrolytic activity of TcTS was indeed due to disruption of the water network by this charged loop, placing the catalytic water in an orientation unfavorable for catalysis [52]. A similar phenomenon has been observed for a native GH31 α-transglucosylase [114].

The second example relies on the results obtained with two GH29B α1,3/4-l-fucosidases. While *Cp*Afc2 from *Clostridium perfringens* efficiently catalyzed transglycosylation, *Bb*AfcB from *Bifidobacterium bifidum* was hampered by high hydrolytic activity [53]. Structural alignment of their homology models as well as sequence alignment revealed that all substrate-interacting residues and the main structural features aligned well. However, *Cp*Afc2 featured a loop approx. 13 Å from the ligand, which in *Bb*AfcB was further away and more open and disordered. Sequence alignment was poor in this region as the two loops differed in length as well as in sequence [54]. It was hypothesized that replacement of the loop sequence in *Bb*AfcB with that of *Cp*Afc2 would improve the transfucosylation ability of *Bb*AfcB through better shielding of the active site from the aqueous environment. Unlike the case for TcTS and TrSA, these loops did obviously not align structurally, and great care was therefore taken to define the starting point and end of the loops to be exchanged. Replacement of a 23-amino-acids-long loop from *Bb*AfcB with the corresponding 17-amino-acid loop of *Cp*Afc2 resulted in almost complete quenching of the hydrolytic activity on 3-FL, while the transfucosylation activity was lowered by only one order of magnitude. As a result, the transfucosylation yield of the loop mutant was comparable to that of *Cp*Afc2 [54].

In order to extract a general strategy from these two examples, it is important to acknowledge the need for a template enzyme from the same GH (sub)family which has a high transglycosylation activity, be it a native transglycosidase or not. In both cases, pathogenic organisms provided these templates. Indeed, pathogenic organisms are, in general, a promising source of transglycosidases, because they use this activity as part of their camouflage strategy [115]. Having found a suitable template, the next step is to identify major differences between the template enzyme and the enzyme to be engineered through both sequence and structural alignments. Targeting areas with large chemical differences or different loop sizes are obvious candidates.

Improving transglycosylation activity of a GH20 β-*N*-acetylhexosaminidase for synthesis of the HMO precursor LNT2 by loop engineering was carried out differently [59]. Since no natural GH20 transglycosidase is known, the structure comparison approach could not be used. As an alternative to identification of beneficial loop sequences and structural placement of those, the bioinformatic tool peptide pattern recognition (PPR) was used [116]. This technique basically performs grouping of a set of protein sequences based on short conserved peptide sequences to identify novel enzymes with potentially new activities [117]. The entire GH20 family as found in CAZy (approx. 3700 sequences) was submitted to PPR analysis, which resulted in 34 different groups. Two previously described β-*N*-acetylhexosaminidases (HEX1 and HEX2) able to synthesize LNT2 from chitobiose and lactose with very low yields [58] were found in the largest group, consisting of approx. 1000 sequences. Detailed phylogenetic tree analysis of this group led to identification of four different five-amino-acid stretches, which were present in GH20 enzymes closely related to HEX1 [59]. Some of these sequences originated from pathogenic bacteria, e.g., the fish pathogen *Renibacterium salmoninarum*, and were thus potentially natural transglycosidases. All additional loop sequences were charged, which was described previously to increase transglycosylation activity [52,74]. Finally, preparation of homology models of the loop-engineered variants revealed that the oxazoline intermediate was more shielded from bulk water with the additional loop stretches present. The transglycosylation activity of eight different loop variants was investigated, and three of them had an increased activity compared to the wild type. The best variant showed a 9-fold higher overall transglycosylation yield [59]. However, four different LNT2 isomers were produced, thus underlining that regioselectivity is probably still the most challenging characteristic to be engineered.

No other examples of loop engineering with the aim of improving transglycosylation by HMO-relevant GHs exist, but examples of loop exchange, deletion, and insertion to alter enzyme specificities exist and may be used for inspiration [118,119,120,121,122,123,124].

### 4.3. Effect of Protein Engineering on Reaction Rates

In most cases of glycosidase engineering for improved transglycosylation yields, the increased T/H is obtained through reduction of the hydrolysis rate rather than through an increase in the transglycosylation rate [50,52,54,74,90]. In fact, the transglycosylation rate usually decreases as well, albeit not as much as the hydrolytic rate [50,54,74]. Frequently, the general enzyme activity decreases with the number of point mutations introduced [67,71,73,75,95,108].

## 5. Abundant Natural Substrates from Dairy and Agro-Industrial Side Streams

A requirement for obtaining a practicable process is the availability of cheap substrates. Microbial cell factories convert simple sugars into HMOs (see Section 2.1), thus keeping the process cost-efficient. For in vitro enzymatic processes, glycosidases hold the largest potential in terms of providing a low-cost process because they accept cheap, naturally available substrates. While activated, synthetic donor substrates often give higher HMO yields due to their good leaving groups, they are exclusively useful for analytical purposes as well as for enzyme discovery and engineering [49,125]—not for industrial processes. The use of lactose is obvious as it is highly abundant in the dairy industry and present as the core of all HMOs (Figure 1). Identification of industrial side streams that are useful donor substrates for HMO production mainly relies on the presence of the relevant monosaccharides (Fuc, Sia, GlcNAc, and Gal) in a terminal position where the GH in question recognizes the unoccupied nonreducing end as well as the linkage to the neighboring moiety. For development of an industrial HMO production process, food-grade streams are preferred. Not all HMO building blocks are equally easy to find in such a setting. This section provides a status on the synthesis of HMO structures from abundantly available agro-industrial side streams.

The most developed process is the use of casein glycomacropeptide (CGMP) as a sialyl donor (Table 1). CGMP is the 64-amino-acids-long C-terminal of κ-casein, which is released into the whey upon chymosin action in cheese manufacturing. This soluble glycoprotein makes up approx. 20% of the whey protein [126]. Whey is produced in massive quantities and CGMP is thus abundantly available and of food-grade quality [3,127]. CGMP has a fairly defined glycosylation pattern, which includes 4–9% Sia in terminal positions [69,128]. The distribution between α2,3- and α2,6-linked Sia is almost even: the percentage of α2,3-linked sialic acid is in the 50–59% range [69,129]. After use as a sialyl donor, the desialylated CGMP is still useful as a protein supplement. Several examples of the use of CGMP as a sialyl donor for synthesis of sialylated HMOs exist [42,48,52,56,66,70,130]. Among the enzymes employed are the native *T. cruzi* transsialidase TcTS, engineered variants of the *T. rangeli* sialidase TrSA, the dual-activity sialyltransferase from *Pasteurella multocida*, and several other microbial sialidases (Table 1). Molar yields on the donor substrate—Sia bound in CGMP—generally ranged from 19–37% for the enzymes specific for α2,3-sialosides (Table 1). Considering that only 50–59% of the Sia bound in CGMP is available for these enzymes, especially the yields in the 30–40% range must be considered high enough to hold industrial potential. For the dual-activity sialyltransferase from *Pasteurella multocida*, which formed both 3′-SL and 6′-SL, the total yield was 53% [70] (Table 1). Membrane filtration setups for up- and downstream processing have been suggested [131,132,133], and performance of the reaction in an enzymatic membrane reactor increased the 3′-SL yield and biocatalytic productivity [134]. Indeed, since both CGMP and Lac are abundantly available dairy streams, the industrial interest in their use for enzymatic synthesis of sialylated oligosaccharides is evident [130,135], and direct application of TcTS in milk and whey for 3′-SL enrichment was patented 20 years ago [136].

Another sialyl donor substrate is polysialic acid, a polymer of α2,8-linked Sia, which is also known as colominic acid. Polysialic acid can be produced by fermentation of engineered *E. coli* and thus has the potential to become a cheap and abundant substrate [138]. In a recent study, polysialic acid was used as a sialyl donor for a sialidase from *Bacteroides fragilis*, which selectively catalyzed formation of α2,6-sialosides: the highest yields of 6′-SL (22%; Table 1) were obtained when polysialic acid was turned into oligosialic acid with a simple acid hydrolysis method ready for industrial application before transglycosylation [57]. Colominic acid has also been used as a sialyl donor for other sialidases, albeit with much lower yields [49]. Another abundant source of sialyl donors is slaughterhouse waste [139]. Bovine blood plasma glycoprotein (BPG) and porcine small intestinal mucin glycoprotein (PSMG) have also been mentioned as sialyl donors for TcTS, but unlike bovine CGMP, they both contain a mixture of two sialic acids, namely *N*-acetylneuraminic acid (Neu5Ac, the one found in HMOs and >99% of the Sia in CGMP) and *N*-glycolylneuraminic acid (Neu5Gc) [135,140]. However, Neu5Gc from mammalian dietary sources can be incorporated into human tissues, where it causes inflammation. Neu5Gc is also believed to play a central role in cancer development, although it may only be carcinogenic in combination with other factors [141,142,143]. Similarly, fetuin from fetal calf serum has been used as a sialyl donor for synthesis of 3′-SL with TcTS in a high-yield reaction (76%; Table 1) [137].

Chitin, a (β1,4)-GlcNAc polymer, is a structural component of fungal, algal, and yeast cell walls and is present in the exoskeleton of crustaceans, mollusks, and insects [144,145]. Chitin is similar to cellulose in terms of both structure and functionality in nature, recalcitrance, and abundance. Indeed, its abundance in the ecosystem is measured in gigatons [144]. The main source of industrial chitin is shellfish waste, which readily grants it food-grade status. Estimates of the amount of chitin-rich shellfish waste are in the megaton range [145]. Chitin extraction from shellfish waste traditionally takes place in two steps (demineralization and deproteinisation), which can be performed either chemically or microbially [146]. However, chitin polymers are insoluble and therefore need depolymerization before chitin can be utilized as a GlcNAc donor for HMO synthesis [147]. Generally, transglycosylation is more efficient when using donor substrates with a low degree of polymerization [53,57]. Such depolymerization could be accomplished by combining chitinases and chitin-active lytic polysaccharide monooxygenases, as done by microorganisms able to degrade the recalcitrant polymer [148,149], preferably as an optimized minimal enzyme cocktail suitable for industrial application [150]. Evidently, the process from shellfish waste to HMOs is still in its infancy, but many of the required steps have been studied separately, e.g., the use of chitobiose, a substrate which could be produced by chitin depolymerization, as a donor substrate in the enzymatic production of the HMO core component LNT2, albeit with yields below 10% [58,59] (Table 1).

Following generation of LNT2 or similar HMO precursor structures, a terminal Gal residue must be added to yield a full HMO structure (Figure 1). This can be accomplished by β-galactosidases, which are already widely used in industry for GOS production [18,19]. Using one of the most efficient commercial β-galactosidase preparations—Biolacta, which contains several *Bacillus circulans* β-galactosidases [151]—a 19% yield of LNnT was obtained from a reaction with Lac and LNT2 [61] (Table 1). A one-pot cascade reaction from Lac to LNnT was not accomplished due to low LNT2 yields [60]. β-Galactosidase-catalyzed production of LNT from LNT2 has only been reported with a *o*-nitrophenyl galactoside donor using a GH35 β1,3-galactosidase from *B. circulans* [61]. An alternative to this two-step approach is the use of lacto-*N*-biosidases, which transfer disaccharides, but so far, this has only been accomplished with a *p*-nitrophenyl-activated donor substrate [61]. In summary, this process can hardly compete with production of LNT and LNnT in microbial cell factories. However, the sequential use of β-*N*-acetylhexosaminidases and β-galactosidases may provide a solution to efficiently obtaining HMOs branched on the Gal moiety of Lac, a type of HMO synthesis reaction which is currently not possible to obtain in microbial cell factories [13].

Suitable, natural Fuc donor substrates useful for HMO production are scarce. However, enzymatic transfucosylation from fucosylated citrus peel xyloglucan to yield 2′-FL was recently reported [53] (Table 1). Product yields were moderate (14% on the donor), and there is room for improvement by protein engineering; by increasing substrate accessibility, e.g., by xyloglucanase treatment; or by looking for other plant sources with higher Fuc content. Abundantly available sources of fucosylated xyloglucan include citrus peel, berry press residues [152], and peanut shells [153,154]. The Fuc-rich polymer fucoidan is found in brown seaweed, but the Fuc units are often sulfated [155]. While a few fucoidan-degrading fucosidases have been described [156,157], no reports of transglycosylation from fucoidan exists. However, the biomass resources are vast, and it is hypothesized that fucoidan can be made accessible as a fucosyl donor through degradation by fucoidanase and sulfatase. Fuc is also present in a terminal position on mucin, which can be isolated from slaughterhouse waste. The α1,3/4-l-fucosidase *Cp*Afc2 from *Clostridium perfringens*, which has considerable transfucosylation activity [53], is hydrolytically active on porcine gastric mucin [158]. However, no reports of transfucosylation with mucin as a fucosyl donor exist. In conclusion, utilization of fucosylated industrial side streams for HMO production is still in its infancy. Alternatively, simple fucosylated compounds produced by microbial cell factories could serve as donor substrates as outlined below.

It is evident that only the use of CGMP and Lac is close to industrial application. Currently, glycosidase-catalyzed synthesis using natural substrates cannot compete with microbial cell factories or chemical synthesis for production of 2′FL at an industrial scale. However, glycosidase-catalyzed transglycosylation may turn out to be a competitive process for more complex HMO structures, because enzyme catalysis is not significantly hampered by small increases in substrate size. While the yields in Table 1 may appear moderate, they provide proof-of-concept of the technology, and protein engineering clearly manifests itself as a valuable tool to provide sufficiently efficient biocatalysts. From here, stepwise process optimization is required to develop a cost-efficient process, e.g., through further enzyme engineering and recycling of enzyme and unreacted substrate. For natural polymeric substrates, some upstream extraction work may be required to help increase the access of the enzyme to the substrate or solubilize the reactive parts of the substrate and hence improve reaction rates and yields. However, enzymatic reactions may require less downstream purification than production by microbial cell factories. Enzymes can be produced on demand: today, industrial-scale expression of recombinant enzymes is already a large business, and especially GHs are generally easy to express in high yields and are robust in operation. Evidently, more work is required before glycosidase-catalyzed synthesis of HMOs reaches the same industrial potential as production of GOS and FOS [18,19,20], but the existence of the enzymatic GOS and FOS production processes provides evidence that enzymatic transglycosylation can be a cost-efficient industrial process.

Current focus is on utilization of smaller HMO structures produced by fermentation or by sialyltransferases as substrates for production of more complex HMOs using glycosidase-catalyzed transglycosylation. Fucosyllactoses 2′-FL and 3-FL and sialyllactoses 3′-SL and 6′-SL, as well as backbone structures LNnT and LNT, are among the few HMOs which are available in large or medium scale [9,159]. For example, sialyllacto-*N*-tetraose c (LST c) was obtained from 6′-SL and LNnT in a reaction catalyzed by the sialidase activity of the dual-activity sialyltransferase from *Photobacterium leiognathi* after extensive protein engineering [160,161]. Another example is the use of the regiospecific GH29B α1,3/4-l-fucosidases—either as a native enzyme from *Clostridium perfringens* or engineered forms from *Bifidobacterium bifidum* or *B. longum* subsp. *infantis*—which led to appreciable yields of LNFP II and LNFP III in reactions with 3-FL and LNT or LNnT, respectively [54,55]. Using an engineered α1,3/4-l-fucosidase with minimal hydrolytic activity [54], such a reaction would yield a mixture of three HMOs (e.g., 3-FL, LNT, and LNFP II) and lactose, which are all relevant for infant formula addition. Together with comprehensive industry-driven engineering studies on the *B. longum* subsp. *infantis* fucosidase to yield other complex fucosylated HMOs [162], these examples indicate the interest in this approach. Indeed, glycoside hydrolases hold the potential to be engineered to widen the current industrial HMO portfolio significantly, using abundant natural substrates as well as simple HMOs produced by other viable methods.

## 6. Conclusions and Perspectives

HMO production by microbial fermentation was shown to be a viable route 20 years ago [9]. After decades of metabolic engineering of *E. coli*, from knock-out of genes encoding for substrate-degrading enzymes to knock-in of genes encoding for relevant glycosyltransferases, alongside several other efforts in expression regulation and transport, it is now possible to produce a handful of HMO structures in industrial scale by fermentation of engineered *E. coli* [9]. However, certain important structures are not possible to obtain by metabolic engineering and fermentation. For instance, the lack of a suitable β-1,6-*N*-acetylglucosaminyltransferase blocks the possibility of obtaining branched HMO compounds [13]. Glycosidase-catalyzed transglycosylation holds the potential of being implemented as an alternative and promising technology which can facilitate diversification of the HMO structures in order to access a more structurally complex and diverse HMO portfolio. Its success as an industrial technology is largely dependent on successful engineering of glycosidases to exhibit significant transglycosylation yields. The protein engineering strategies used to design HMO molecules may prove useful for other enzymatic carbohydrate synthesis reactions in the future.

## Figures and Tables

**Table 1:** Synthesis of true HMO structures where abundantly available natural substrates—or derivatives of these—have been employed. Yields are given as molar yield based on the donor substrate (for casein glycomacropeptide (CGMP), all available Sia moieties are considered, although not all are present with a linkage accepted by the enzymes; see the main text). Abbreviations: A:D: molar donor-to-acceptor ratio; n.d.: not determined.

Enzyme	Donor	Acceptor	HMO product	A:D	Yield	Ref.
*Arthrobacter ureafaciens* sialidase	CGMP	Lac	n.d.	~45	5%	[130]
*Bifidobacterium infantis* sialidase	CGMP	Lac	n.d.	~45	1%	[130]
*Trypanosoma rangeli* sialidase (engineered)	CGMP	Lac	3′-SL	44	31%	[52]
*Trypanosoma rangeli* sialidase (engineered, membrane reactor)	CGMP	Lac	3′-SL	≤25	37%	[134]
*Pasteurella multocida* sialidase (sialyltransferase)	CGMP	Lac	6′-SL, 3′-SL	11	53%	[42,70]
*Haemophilus parasuis* sialidase	CGMP	Lac	3′-SL (and isomer)	39	19% (28%)	[66]
*Trypanosoma cruzi* transsialidase	CGMP	Lac	3′-SL	5	32%	[48]
*Trypanosoma cruzi* transsialidase	Fetuin	Lac	3′-SL	3	76%	[137]
*Bacteroides fragilis* sialidase	Hydrolyzed colominic acid	Lac	6′-SL	~15	22%	[57]
*Fusarium graminearum* fucosidase	Citrus peel xyloglucan	Lac	2′-FL	50	14%	[53]
β-*N*-acetylhexosaminidases (engineered, metagenomic)	Chitobiose	Lac	LNT2 (and isomers)	5	5% (30%)	[59]
*Bacillus circulans* β-galactosidases	Lac	LNT2	LNnT	1	19%	[61]
